# Association Between *Trypanosoma cruzi* DNA in Peripheral Blood and Chronic Chagasic Cardiomyopathy: A Systematic Review

**DOI:** 10.3389/fcvm.2021.787214

**Published:** 2022-01-31

**Authors:** Pau Bosch-Nicolau, Juan Espinosa-Pereiro, Fernando Salvador, Adrián Sánchez-Montalvá, Israel Molina

**Affiliations:** ^1^Tropical Medicine & International Health Unit Vall d'Hebrón - Drassanes, Infectious Diseases Department, PROSICS Barcelona, University Hospital Vall d'Hebron, Barcelona, Spain; ^2^Medicine Department, Universitat Autònoma de Barcelona, Barcelona, Spain

**Keywords:** Chagas disease, Chagas cardiomyopathy, *T. cruzi*, polymerase chain reaction, PCR

## Abstract

Chronic chagasic cardiomyopathy (CCC) is the most important complication of patients with Chagas disease (CD). The role of persistent detection of DNA in peripheral blood and its association to CCC is unknown. We performed a systematic review up to July 2021, including studies that reported ratios of CCC and PCR positivity among non-treated adult patients. We identified 749 records and selected 12 for inclusion corresponding to 1,686 patients. Eight studies were performed in endemic countries and 4 in non-endemic countries. Only two studies showed an association between CCC and *Trypanosoma cruzi* parasitemia by means of PCR detection. Six studies reported greater positive PCR ratios among patients with CCC than in the patients with indeterminate chagas disease (ICD) with no statistical significance. A significant risk of bias has been detected among most of the studies. Therefore, while we performed a meta-analysis, wide inter-study heterogeneity impeded its interpretation.

**Conclusions:**

With the available information, we could not establish a correlation between PCR-detectable parasitemia and CCC.

**Systematic Review Registration:** https://www.crd.york.ac.uk/prospero/display_record.php?ID=CRD42020216072, identifier: CRD42020216072.

## Introduction

Chagas disease (CD) is a protozoal disease caused by *Trypanosoma cruzi*, a zoonotic infection mainly found in endemic areas of the American continent. It affects about 8 million people worldwide and because of globalization and international migrations during the last decades, it has become a cause of concern in non-endemic countries (1).

Chagas disease has an acute phase that generally runs its course asymptomatic or with rather unspecific symptomatology. Once patients overcome this phase, they enter a chronic phase, defined by the absence of trypomastigotes in the blood smear. Most people with *T. cruzi* infection are diagnosed at this stage. Approximately 30–40% of chronically infected patients will develop visceral involvement comprising the chronic chagasic cardiomyopathy (CCC), the digestive form, or both during the following 10–30 years after infection (2). CCC is considered the cause of at least 7,000 deaths every year and is the most common reason for performing heart transplants in Latin America. The principal underlying causes are sudden death from malignant arrhythmias and heart failure as a consequence of dilated cardiomyopathy (3).

Both host immune response and the persistence of infection are crucial on CCC progression (4). Host factors, such as genetic polymorphisms involved in the immune response have been proposed as prognosis markers (5). Regarding the role of *T. cruzi* persistence, several studies have evidenced that visceral involvement is directly linked to the parasite presence on such organs in both human and animal models (6). Besides, an association between certain discrete typing units (DTU), which are used to group *T. cruzi* genetic diversity, and its virulence and tissue tropism has been described (7).

Blood parasites can be detected by PCR. However, parasite dynamics is still a matter of debate. While in the acute phase, the presence of *T. cruzi* in the blood is constant, in the chronic phase low level parasitemia is observed in a subset of patients (8). Different PCR assays have been developed to detect the parasite DNA with initial difficulties in standardizing techniques to obtain reliable and comparable results (9). However, in recent years, notable advances have been made with new methodologies showing reliable results that have been used to monitor the parasite load in patients with CCC and follow-up the effectiveness of etiologic treatments (10). Besides, the relationship between parasitemia or the presence of parasitic DNA on peripheral blood, and disease progression is controversial (11, 12).

Therefore, this systematic review has the objective of assessing the association between the presence of parasitic DNA of *T. cruzi* on peripheral blood through PCR and CCC.

## Materials and Methods

### Study Design

We conducted a systematic review following the standardized guidance (13), and we adhered to the Preferred Reporting Items for Systematic Reviews and Meta-Analyses (PRISMA) statement using a flow diagram and following its checklist to ensure that all recommended information is captured and findings are properly reported (14). The review protocol was registered in the PROSPERO database (registration ID number: CRD42020216072).

### Eligibility Criteria and Patient Population

We included clinical trials, controlled observational and cross-sectional studies in adult patients (>16 years old) with chronic CD reporting data on the results of the peripheral blood *T. cruzi* PCR and CCC.

Eligible studies had to establish the chronic CD diagnosis through two different positive serological tests. Indeterminate Chagas disease (ICD) concerns patients diagnosed with a chronic CD that presented with normal ECG and/or echocardiogram, regardless of the presence or absence of gastrointestinal disease. CCC was defined as electrocardiographic or echocardiographic alterations not attributed to other conditions. We included studies considering any PCR protocol whenever the same procedure was maintained throughout the study.

Studies assessing the impact of treatment with benznidazole, nifurtimox, or azole-derivative drugs were excluded. In addition, we excluded studies focusing on acute infections, pregnant women, children, or immunocompromised patients.

### Literature Search, Data Collection, and Reporting of Results

We searched Medline, EMBASE, and LILACS databases. Additionally, we tracked citations to relevant studies in Scopus and the ISI Web of Knowledge for review purposes, and manually screened references lists of these studies. We adapted the search strategy to the requirements of each database (Supplementary Material Appendix 1). There were no language or publication period restrictions. We conducted the last search during July 2021.

One reviewer (PBN) screened the titles and abstracts resulting from the search against inclusion criteria. We obtained a full-text copy of eligible references to finally decide on their inclusion. A second reviewer (JEP) independently checked the eligibility decisions for accuracy. We discussed disagreements until a decision was reached; and planned to involve a third investigator if discrepancies remained.

Two researchers (PBN and JEP) extracted data from included studies using the standardized extraction forms. No pilot study was conducted due to the low number of articles included. Whenever possible, article authors were contacted for unreported or additional data to minimize the missing data. For each study, we collected (when available): study design, baseline characteristics, cardiac involvement, PCR method, PCR status of the included patients at the inclusion, and association measures as reported by authors of each study. We independently assessed the risk of bias for each study using a modified Quality In Prognosis Studies (QUIPS). Tool checklist (Supplementary Material Appendix 2), appropriate for prognostic factor review questions (15).

### Statistical Analysis

We described the population of each study and the frequencies of positive PCR among ICD and CCC participants. The association of PCR status and visceral involvement was extracted as the amount of positive PCR among ICD participants and CCC participants, using as association measure the odds ratios (ORs) and 95% *CI*. Pooled *OR* was calculated using the Mantel–Haenszel approach with a random effects model due to the heterogeneity of each study population. Inter-study heterogeneity was assessed with a restricted maximum likelihood model and reported as the *I*^2^ and tau statistics. We adhered to the methods recommended in the Cochrane Handbook for Systematic Reviews of interventions whenever possible (16).

## Results

**Figure 1:**
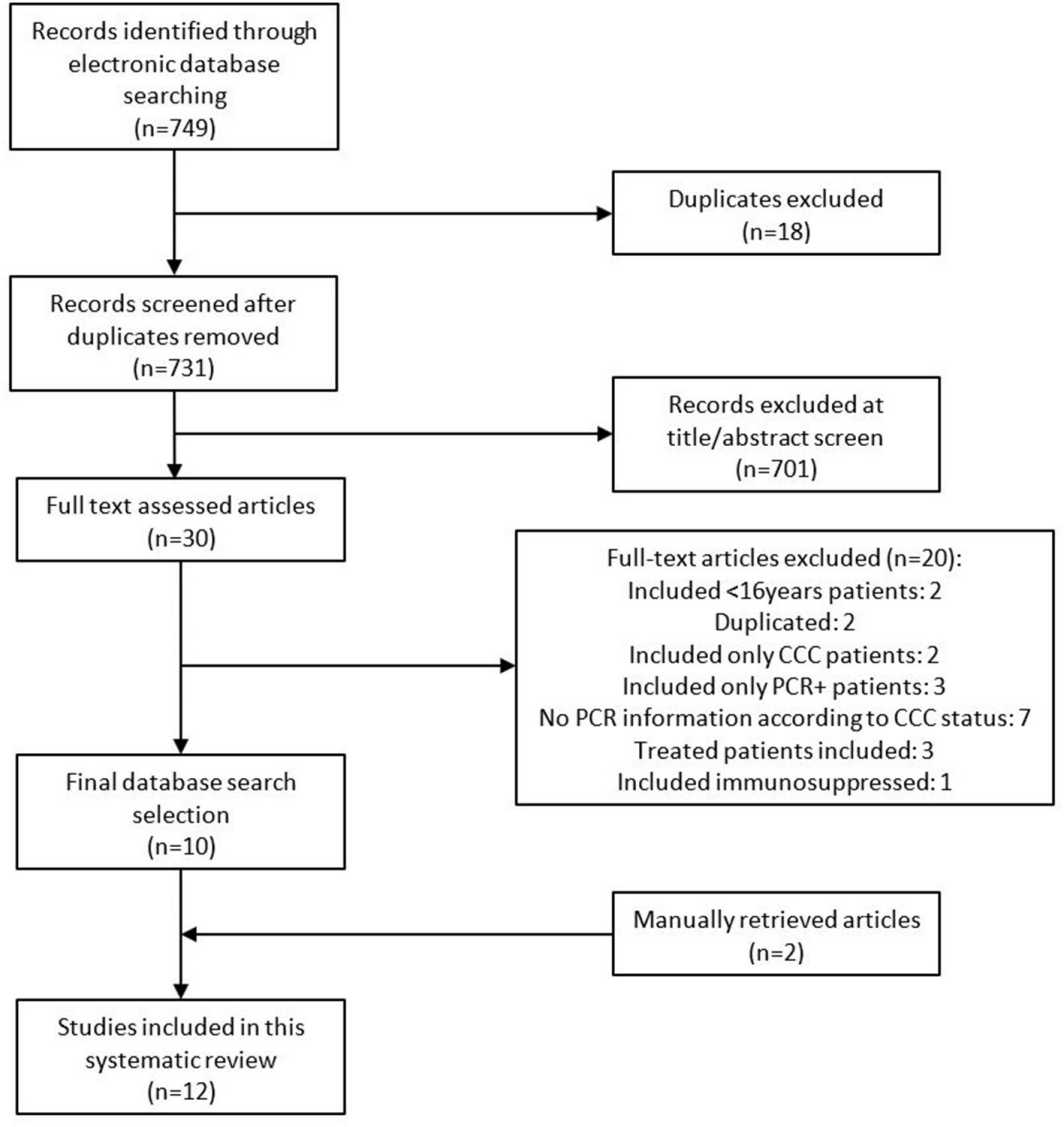
The Preferred Reporting Items for Systematic Reviews and Meta-Analyses (PRISMA) flow diagram of included articles. CCC, chronic chagasic cardiomyopathy; PCR, polymerase chain reaction.

Our search retrieved 749 records. After removing duplicates and reading titles and abstracts, we discarded 719 records as they were *in vitro* experiments, were performed in animals, corresponded to *Trypanosoma* species other than *T. cruzi*, included acute infection patients or treated patients, or did not report visceral involvement. Of the remaining 30 reports, we proceeded to a full-text review and finally included 10 articles. The characteristics of excluded studies are reported in Supplementary Material Appendix 3 (8, 11, 17–34). After checking the reference lists of these studies, we identified two additional eligible studies. The complete eligibility process is depicted in a PRISMA flowchart (Figure 1).

Finally, we included 12 studies comprising 1,686 patients (12, 35–45), which are described in Table 1. Eight studies were cross-sectional studies, 3 were designed as prospective cohorts, and 1 was a case-control study. Countries where studies were performed in endemic areas were as follows: 3/8 (37.5%) in Brazil, 3/8 (37.5%) in Chile, 1 (12.5%) in Argentina, and 1 (12.5%) in Bolivia. Among them, 1 was performed in a rural site, 2 in urban facilities, and 5 combined patients living in both urban and rural contexts. Four studies included patients from non-endemic countries: 3 from Spain where more than 90% of patients came from Bolivia and 1 from Japan. Patients' ages ranged from 16 to 81 years old, and most of them were women (58.8%; 885/1,505).

**Table 1:** Summary of the characteristics of the included studies.

**References and country**	**Study design**	**Study site**	**Included (excluded)**	**Age**	**Sex**	**Excluded comorbidities**	**CCC assessment**	**Clinical form**	PCR technique*	**PCR results**	**Association measure**
Salomone et al. (35) Argentina	Cross-sectional	Endemic (urban)	68 (23)	55 y (SD ± 12) ICD: 52 y (SD ± 9) CCC: 59 y (SD ± 12)	64% F ICD: 70% F CCC: 59% F	Yes	EKG + EchoC	ICD: 27 (39.7%) CCC: 41 (60.3%)	Qualitative c-PCR 1 sample Kinetoplast DNA	Global + PCR: 14/68 (21%) ICD: 2/27 (7.4%) CCC: 12/41 (29.2%)	OR 5.17 (95%CI 1.06–25.36)
Carrasco et al. (36) Chile	Cross-sectional	Endemic (rural)	38 (225)	ICD: 40,57 y (SD ± 10.59) CCC: –HF 68.4 y (SD ± 12.9) –PM: 54.1 y (SD ± 8.4) –Altered EKG: 53.4 y (SD ± 19.1)	ICD: 62% M CCC: 62% M	No	EKG + EchoC + Thoracic X-Ray	ICD: 26 (68.4%) CCC: 12 (31.6%)	Qualitative c-PCR 1 sample Kinetoplast DNA	Global + PCR: 18/38 (54.7%) ICD: 10/26 (36%) CCC: 8/12 (66%)	OR 3.20 (95%CI 0.76–13.46)
Zulantay et al. (37) Chile	Prospective cohort	Endemic (urban and rural)	30 (0)	33.2 y (R 18–50)	53.3% M	Yes	EKG	ICD: 18 (60%) CCC: 12 (40%)	Qualitative c-PCR 1 sample Kinetoplast DNA	Global + PCR: 17/30 (56.6%) ICD: 10/18 (55.5%) CCC: 7/12 (58.3%)	OR 1.12 (95%CI 0.26–4.91)
Borges-Pereira et al. (38) Brazil	Cross-sectional	Endemic (rural)	12 (9)	48.6 y (R 16–82) ICD: 43.5 y CCC: 64.4 y	58,8% F ICD: 70% F CCC: 42.9% F	No	EKG	ICD: 6 (50%) CCC: 6 (50%)	Qualitative c-PCR 1 sample Kinetoplast DNA	Global + PCR: 9/12 (75%) ICD: 5/6 (83.3%) CCC: 4/6 (66.6%)	OR 0.40 (95%CI 0.03–6.18)
Murcia et al. (39) Spain	Prospective cohort	Non endemic	181 (0)	33 y (SD ± 11)	No reported	No reported	EKG + EchoC + Thoracic X-Ray	ICD: 116 (64%) CCC: 65 (36%)	Qualitative c-PCR 1 sample Kinetoplast DNA	Global + PCRs: 123/181 (68%) ICD: 81/116 (69,8%) CCC: 42/65 (64.6%)	OR 0.79 (95%CI 0.41–1.50)
Sabino et al. (12) Brazil	Cross-sectional	Endemic (urban and rural)	485 (115)	IND (PCR–): 48.4 y (SD ± 10.1) IND (PCR+): 49.1 y (SD ± 10.6) CCC (PCR–): 49.2 y (SD ± 6.3) CCC (PCR+): 47.8 y (SD ± 7.1)	53,6% M IND: 52,3% M CCC: 59,4% M	Yes	EKG + EchoC	ICD: 279 (57.5%) CCC: 206 (42.5%)	Quantitative rt-PCR 1 sample Kinetoplast DNA	Global + PCR: 304/485 (62.7%) ICD: 143/279 (51.3%) CCC: 161/206 (78.1%)	OR 3.48 (95%CI 2.31–5.23)
Kaplinski et al. (40) Bolivia	Cross-sectional	Endemic (urban and rural)	83 (337)	ICD: 27 y (R 22–34) CCC: 32 y (R 24–39)	100% F	No reported	EKG	IND: 73 (87.9%) CCC: 10 (12.1%)	Quantitative rt-PCR 1 sample Kinetoplast DNA	Global + PCR: 36/83 (43.4%) ICD: 33/73 (45.2%) CCC: 3/10 (33.3%)	OR 0.52 (95%CI 0.12–2.17)
Apt et al. (41) Chile	Case-control	Endemic (urban and rural)	200 (0)	ICD: 50.5 y (R 20–77) CCC: 56.4 y (R 25–81)	ICD: 79% F CCC: 68% F	Yes	EKG + EchoC	ICD: 100 (50%) CCC: 100 (50%)	Qualitative c-PCR 1 sample Kinetoplast DNA	Global +PCR: 145/200 (72.5%) ICD: 72/100 (72%) CCC: 73/100 (73%)	OR 1.05 (95%CI 0.57–1.96)
Sánchez-Montalvá et al. (45) Spain	Cross-sectional	Non endemic	455 (316)	39 y (R 31–46.5) ICD: 37 y (R 31–44) CCC: 42 y (R 36–49)	68.2% F	Yes	EKG + EchoC + Thoracic X-Ray	ICD: 302 (66.4%) CCC: 153 (43.6%)	Qualitative rt-PCR 1 sample Satellite DNA	Global +PCR: 118/455 (25.9%) ICD: 76/302 (25.2%) CCC: 42/153 (27.4%)	OR 1.13 (95%CI 0.72–1.75)
D'Ávila et al. (42) Brazil	Cross-sectional	Endemic (urban and rural)	91 (0)	ICD: 44 y (SD ± 10.3) CCC: 54 y (SD ± 10.3)	ICD: 65.2% F CCC: 33.8% F	No reported	EKG + EchoC + Thoracic X-Ray	ICD: 23 (33.8%) CCC: 68 (66.2%)	Quantitative rt-PCR 1 sample Satellite DNA	Global + PCR: 65/91 (71.4%) ICD: 16/23 (69.6%) CCC: 49/68 (72%)	OR 1.13 (95%CI 0.40–3.17)
Salvador et al. (44) Spain	Prospective cohort	Non endemic	38 (16)	36 y (R 22–55)	75.6% F	No reported	EKG + Thoracic X-Ray	ICD: 27 (71%) CCC: 11 (29%)	Qualitative rt-PCR 1 sample Satellite DNA	Global + PCR: 16/38 (42.1%) ICD: 11/27 (40.7%) CCC: 5/11 (45.4%)	OR 1.21 (95%CI 0.29–4.98)
Imai et al. (43) Japan	Cross-sectional	Non endemic	5 (12)	57.6 y (R 49–68)	60% F	No	EKG + EchoC	ICD: 1 (20%) CCC: 4 (80%)	Qualitative rt-PCR 1 sample Satellite DNA	Global +PCR: 3/5 (60%) ICD: 1/1 (100%) CCC: 2/4 (50%)	OR 0.33 (95%CI 0.01–12.82)

*CCC, chronic chagasic cardiomyopathy; c-PCR, conventional polymerase chain reaction; EchoC, echocardiography; ECG, electrocardiogram; ICD, indeterminate chagas disease; R, range; RR, risk ratio; rt-PCR, real time polymerase chain reaction; SD, standard deviation; y, years; PM, pacemaker; HF, heart failure*.

* *PCR methodology: specifying PCR technique (conventional PCR or real time PCR) and target of the used primer (kinetoplast DNA or nuclear satellite DNA)*.

Chronic chagasic cardiomyopathy assessment was performed using ECG in all studies. In 4 (33.3%) studies, ECG was combined with echocardiography, in 1 (8%) was combined with chest radiography, and in 4 (33.3%), the 3 ancillary tests were performed. CCC classification was very heterogeneous and included either predefined criteria by the authors (35–37, 40, 43) or standardized classifications as Minnesota criteria (12, 38), Kuschnir criteria (39, 44, 45), Rocha criteria (42), and New York Heart Association (NYHA) classification (41). Among the included patients, 998 (59.2%) were classified as ICD and 688 (40.8%) as CCC. CCC proportion among different studies varied from 12.1% (40) in a study including young childbearing-aged women to 80% (43) in a study including patients under the suspicion of organ involvement in Latin-American people living in Japan. However, the last study included only 5 patients. Cardiologic characteristics of patients with CCC are summarized in Supplementary Material Appendix 4.

All the included studies used a PCR for DNA parasite detection in peripheral blood. However, many different protocols were used for its determination. All studies used a single venous blood sample for PCR determination. Six studies (50%) used a conventional PCR method (35–39, 41) while the other half used real-time PCR method (12, 40, 42–45). Only four studies used a quantitative method to report PCR results (12, 40–42) while the rest of the studies used a qualitative method. There is a wide variation regarding primers used. Most studies (8/12; 66.3% used a real time PCR method based on the amplification of a genomic DNA sequence of the Kinetoplast. In the other 4 studies, satellite DNA amplification was used (42–45). Parasite detection in patients with ICD ranged from 7.4 (35) to 100% (43). When considering patients with CCC, parasite detection varied from 27.4 (45) to 78.1% (12).

The results of the risk of bias assessment using the QUIPS scale are shown in Supplementary Material Appendixes 5, 6. Most studies included a representative population with CD. However, 2 studies were considered at an overall high risk as one included only childbearing-aged women (40), and the other included patients under suspicion of organ involvement (43). As per study attrition, most of the studies were classified as low risk of bias since basal characteristics including PCR and organ assessment were performed at the inclusion and, in consequence, data were available for all participants included in the studies. Regarding outcome measurement (PCR), all the studies specified their protocol and almost all included control samples and maintained the same procedure in all samples. As per CCC assessment, six studies were rated at low risk of bias since they included blind ECG assessment and/or double assessment by different investigators (12, 37, 38, 40, 41, 45). Conversely, two studies that neither used a standardized classification nor reported their ECG or echocardiographic findings were considered at high risk of bias (35, 43). Considering study confounders, all the studies but one was rated as having a moderate to high risk of bias. Most of the studies did not consider cardiovascular risk factors and other possible heart diseases or performed a stratified data analysis. Finally, result presentation and statistical analysis were classified at a moderate to high risk of bias in a wide group of studies. Most of them were designed for another purpose and we retrieved the specific information from their results.

An association between CCC and *T. cruzi* parasitemia by means of PCR detection was found in 2 studies (12, 35). They all were performed in endemic regions and found a greater PCR positivity between patients with CCC and ICD with *OR*s of 5.17 (*CI* 1.06–25.36) and 3.48 (*CI* 2.31–5.23). In one study, although a risk ratio (RR) of 4.45 was reported, it included both cardiac and digestive forms on the analysis, and when *OR* was calculated with only CCC patients, we could not achieve statistical significance (36). None of the non-endemic studies identified an association between parasite DNA detection in peripheral blood and CCC. Six studies reported greater positive PCR ratios among patients with CCC than in patients with ICD although differences did not achieve statistical significance (36, 37, 41, 42, 44, 45). The remaining 4 studies reported a positive PCR rate that favored patients with ICD (38–40, 43).

**Figure 2:**
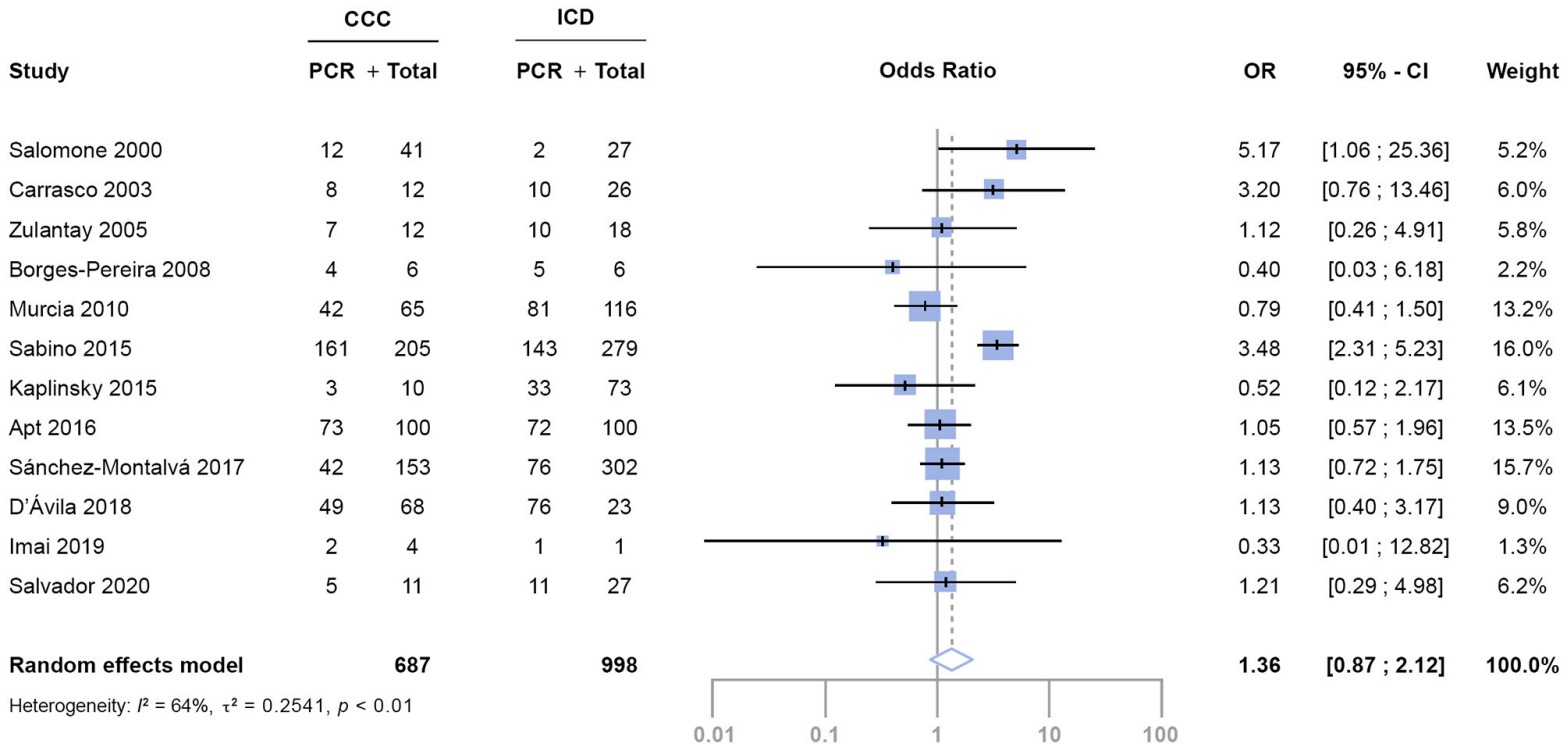
Evaluation of the association between PCR detection between patients with CCC and ICD. CI, confidence interval; OR, odds ratio.

When we meta-analyzed the *OR*, pooled results showed that the estimated *OR* for positive *T. cruzi* PCR of patients with CCC compared to patients with ICD was 1.36 (95% *CI*: 0.87–2.12) *(*Figure 2*)*. However, we found significant heterogeneity among studied variables in the meta-regression analysis. To diminish variability, we grouped studies by patient's countries of origin and by PCR technique with the same results. Thus, no conclusions can be achieved from the meta-analysis.

## Discussion

In this review, despite 8 out of 12 studies reporting higher ratios of positive PCR among patients with CCC, we could not find a correlation between parasitemia by means of PCR and CCC. When we analyzed the characteristics of the included studies, in only 2 studies, the proportion of PCR positivity was significantly greater within CCC (12, 35). These studies were performed in an endemic region and their specific objective was to determine the correlation between parasitemia and CCC. It has been suggested that patients in endemic regions are prone to parasite re-exposure, contributing to higher rates of CCC and parasitemia among patients with CD (45). In the same line, in a study performed in a non-endemic country, the positive PCR ratio decreased in patients with longer periods since the first arrival (8). Also, geographic distribution should play a role in CCC development and parasitemia burden. *T. cruzi* genetic diversity is unequally distributed among different countries, and some authors have reported a cardiac tropism of some discrete typing units (DTUs) (7). In this review, included patients came mostly from 4 different countries and only one of them reported DTUs determination (42).

Age was higher among patients with CCC in studies performed in endemic countries recruiting the general population (range 49.2–68 years) than in non-endemic countries (range 33–42 years). Regardless of the age *per se* having been described as a risk factor for CCC and its mortality, results from different reports are inconsistent (46). On one hand, study participants in their second to the fourth decade of life may have not yet developed target organ damage, or it is too incipient, as it takes on average 20–30 years after the acute infection. On the other hand, older people often associate other cardiovascular risk factors and this could lead to a misinterpretation of the CCC assessment (47). In fact, only three studies were excluded from their analysis due to other cardiovascular risk factors (12, 41, 45). Male sex has been related to CCC development and higher mortality rates (48). Although, this predisposition was described as an independent risk factor from parasitemia (20). Among the included studies, the male proportion ranged from 31.8 to 66.2%. As none of the included studies conducted stratified analysis by sex or age, results should be interpreted with caution.

Classically, CCC assessment has involved ECF, chest X-ray, and clinical status, as they are easy to collect and widely available. Echography, a non-invasive examination, has been progressively studied in CD and its performance has been introduced in different CCC classifications (49). ECG abnormalities are usually seen before the patient develops a malignant arrhythmia or heart failure (50). However, echocardiographic alterations as diastolic dysfunction can appear before electrical abnormalities develop and may be used as an early marker of CCC (45). In this review, the CCC assessment was very heterogeneous. While 3 studies performed only an ECG and 1 study included a chest X-ray, 8 of 12 studies used echocardiography to complete the cardiac evaluation. CCC classifications were disparate, while 5 authors used a non-standardized classification (35–37, 40, 43), 7 used different standardized criteria (12, 38, 39, 41, 42, 44, 45). Conduction system alterations as right-bundle block and left anterior hemiblock prevailed over other arrhythmias or contraction abnormalities in most of the studies (Supplementary Material Appendix 3). Two studies (39, 42) did not provide cardiological findings of their participants, and only 4 studies (41, 42, 44, 45) provided the distribution of disease severity. As a result, between-study comparisons of CCC severity are not feasible.

Current PCR-based methods for *T. cruzi* detection have shown high sensitivity and specificity compared with classical methods, such as blood culture or xenodiagnosis (8). However, sample collection, conservation, sample volume, DNA extraction method, and primers used may influence its performance (9). As a result, in recent years, many initiatives have pursued the standardization of these techniques (33). In the present review, earlier studies used conventional PCR methods preferably using as molecular targets of the kinetoplast DNA sequences. On the other hand, later studies preferred real-time PCR (rt-PCR) techniques toward satellite or minicircle DNA sequences which showed better sensitivity and specificity results and allowed quantification (9). Only four studies used a quantitative method (12, 40–42) but parasitemia levels were not related to any clinical outcome. Parasitemia levels are usually low in chronic patients with CD compared with parasitemia levels in patients with acute CD. Different studies have analyzed the correlation between quantitative results of *T. cruz*i in peripheral blood and organ damage with inconsistent results (41, 50). Parasite detection ratios ranged widely within the included studies. In patients with ICD, the detection ranged from 7.4 (35) to 100% (43) while in patients with CCC varied from 27.4 (45) to 78.1% (12). Besides the previously described factors, parasite dynamics in blood have not been thoroughly characterized. Intermittent parasitemia is constant in the chronic phase of CD but its periodicity, triggers, determinants, or reservoirs are still unknown (8). Thus, although the internal variability is probably acceptable among all studies as they used the same method throughout, lack of sensitivity could result in low positive ratios precluding differences detection.

This systematic review has several limitations. As a neglected tropical disease, scarce investment over time has limited the generation of high-quality evidence (51). A significant risk of bias has been detected among most of the studies resulting from small sample sizes, differences among study designs, differences not reporting on basal characteristics, different CCC assessments, and PCR methods along with the fact that most of the studies were not even designed for this purpose. To improve studies comparability, we have grouped them by the main confounding factors: country of origin and quality of PCR technique but variability remained. Therefore, while we have performed a meta-analysis, wide inter-study heterogeneity (*I*^2^ 64%, *p* < 0.01) impedes a robust interpretation of its results. A patient-level meta-analysis could improve the consistency and robustness of the results and provide evidence to better inform physicians treating people living with CD.

In conclusion, with the current information, we could not establish a correlation between PCR-detectable parasitemia and CCC. Better prospective studies with a defined follow-up, representative population, homogeneous criteria, and standardized methods for PCR detection are needed to answer this question.

## Data Availability Statement

The original contributions presented in the study are included in the article/Supplementary Material, further inquiries can be directed to the corresponding author/s.

## Author Contributions

PB-N has majorly contributed to this study through study designing, abstract and articles revision, data analysis, and manuscript writing. JE-P has contributed to this study through abstract and article revision and final article review. FS, AS-M, and IM contributed through study design and article reviewing. All authors contributed to the article and approved the submitted version.

## Funding

AS-M was supported by a postdoctoral grant Juan Rodés (JE18/00022) from the Instituto de Salud Carlos III through the Spanish Ministry of Economy and Competitiveness.

## Conflict of Interest

The authors declare that the research was conducted in the absence of any commercial or financial relationships that could be construed as a potential conflict of interest.

## Publisher's Note

All claims expressed in this article are solely those of the authors and do not necessarily represent those of their affiliated organizations, or those of the publisher, the editors and the reviewers. Any product that may be evaluated in this article, or claim that may be made by its manufacturer, is not guaranteed or endorsed by the publisher.
