# Supplementary material for: Association Between Trypanosoma cruzi DNA in Peripheral Blood and Chronic Chagasic Cardiomyopathy: A Systematic Review
Source: Front Cardiovasc Med. 2022 Jan 31;8:787214. doi: 10.3389/fcvm.2021.787214 (PMC8841718; doi:10.3389/fcvm.2021.787214)
Supplement: Supplementary file 1 [file Data_Sheet_1.docx]

Supplementary Material

- Appendix 1: EMBASE Search strategy
- Appendix 2: Modified QUIPS tool
- Appendix 3: Characteristics of excluded studies
- Appendix 4: Cardiologic characteristics of CCC patients
- Appendix 5: QUIPS summary
- Appendix 6: Detailed QUIPS assessment by study

**Appendix 1: EMBASE Search strategy**

| No. | Query | Results |
| --- | --- | --- |
| #12  #11  #10  # 9  #8  #7  #6  #5  #4  #3  #2  #1 | #6 AND #11  #7 OR #8 OR #9 OR #10  Pcr:ti  (polymerase NEXT/2 chain NEXT/2 reaction):ti,ab  ‘polymerase chain reaction’/exp/mj  ‘trypanosoma test kit’/exp  #1 OR #2 OR #3 OR #4 OR #5  (t NEXT/1 cruzi):ti,ab  (trypanosoma NEXT/1 cruzi):ti,ab  Chagas:ti,ab  ‘trypanosoma cruzi’/exp  ‘chagas disease’/exp | 749  325,222  54,257  275,104  55,701  21  28,135  9,855  15,706  15,398  15,844  17,005 |

Search results corresponding to 1^st^ July 2021

**Appendix 2: Modified QUIPS tool:**

Here we present a modified QUIPS tool according for this prognostic factor review.

This tool allows reporting those aspects for assessing risk of bias for a study within six domains, to inform the overall potential bias for each domain. According to it, we will rate potential risk of bias as Low, Moderate or High, considering all relevant issues.

**Study participation**: the study sample is representative enough of the population of interest considering those relevant characteristics that limit risk of bias of the observed relationship between the outcome and the prognostic factor.

- Method used to identify the population are adequately described, including sample recruitment method (patients attending a specific clinic, mass population screening).
- Recruitment period is adequately described
- Place of recruitment includes setting and geographic location
- Inclusion and exclusion criteria are adequately described including an appropriate case definition.
- Study participating is adequate among eligible individuals
- Baseline characteristics are adequately described including age, sex, other cardiovascular comorbidities, immunosuppression, or treatment history.

**Study attrition:** Loss of patients is not associated with main characteristics to limit potential bias to the relation between CCC and PCR status.

- Proportion of baseline sample available for analysis is adequate
- Attempts to collect information of lost participants are described
- Reasons of this loss are well reported
- Participant characteristics of those who were dropped out are adequately described (age, sex and cardiac involvement status)

**Prognostic factor measurement:** PCR measurement is adequately performed in individuals to sufficiently limit potential bias.

- Measurement description is provided including number of samples obtained for its assessment, moment of assessment and sample conservation methods.
- Method of PCR is well reported, and a reference or description of the method is provided.
- PCR classification considering qualitative or quantitative results is well specified and cut-off values are reported.

**Outcome measurement:** CCC assessment in the study comprises validated methods and a specific classification is provided to sufficiently limit potential bias.

- Definition of the outcome is clearly provided including EKG results, echocardiography findings and clinical status if available.
- The method of measurement used is adequately valid and reliable to limit misclassifications.
- The method and classification used is an internationally validated scale and is the same for all study participants.

**Study confounding:** important potential confounders as other cardiac involvements, cardiac risk factors and age are appropriately accounted for, limiting potential bias between PCR status and CCC.

- Al important confounders are considered including individual demographics (age or sex), place of residence (urban or rural settings) and cardiovascular risk factors.
- Clear definitions of the confounders are provided
- The method of confounding assessment is the same for all study participants
- Important potential confounders are accounted for in the study design and analysis (matching for key variables, stratification, adjustment for cardiac involvement severity)

**Statistical analysis and reporting:** they are appropriate for the design of the study limiting potential for invalid results and selective reporting is unlikely.

- There is a good presentation of the data to assess the validity of the analysis.
- The selected statistical model is adequate for the design of the study
- There is no selective reporting of the results

**Appendix 3: Characteristics of excluded studies**

| **Study** | **Reason for exclusion** |
| --- | --- |
| Bernière 2002 | Children included |
| Borges-Pereira 2002 | Children included. No information of PCR status depending on visceral involvement. Negative serology patients included. |
| Marcon 2002 | No information of PCR status depending on visceral involvement |
| Basquieira 2003 | All included patients were diagnosed as CCC |
| Pompilio 2005 | Non-Chagasic patients included. No information of PCR status depending on visceral involvement |
| Hidron 2010 | No information of PCR status depending on visceral involvement |
| Llaguno 2011 | Only included PCR positive patients |
| Teixeira de Freitas 2011 | Only immunosuppressed patients included |
| Norman 2011 | Patients with previous treatment were included |
| Ballinas-Verdugo 2011 | Only included PCR positive patients |
| Ramos 2012 | No information of PCR status depending on visceral involvement. Immunosuppressed patients were included. |
| Saavedra 2013 | PCR was performed on xenodiagnoses samples |
| Gilber 2013 | No information of PCR status depending on visceral involvement. Children included. |
| Melo 2015 | Only included PCR positive patients |
| Pires-Antunes 2016 | Treated patients included. No information of PCR status depending on visceral involvement |
| Barroso-Pereira 2016 | No information of PCR status depending on visceral involvement |
| Zempulski-Volpato 2017 | Same patients included in the study by D’Ávila et al. |
| Sulleiro 2020 | Same patients included in the study by Sánchez-Montalvá et al. |
| Buss 2020 | No information of PCR status depending on visceral involvement |
| Sulleiro 2020 | Same patients included in the study by Sánchez-Montalvá et al. No information of PCR status depending on visceral involvement. |

Abbreviations: CCC: chronic chagasic cardiomyopathy; PCR: polymerase chain reaction.

**Appendix 4: Cardiological characteristics of CCC patients.**

| **Reference and country** | **CCC assessment** | **Criteria used** | **CCC severity of included patients** | **CCC findings** |
| --- | --- | --- | --- | --- |
| **Salomone,** O.A. Et al. 2000 Argentina | EKG + EchoC + Thoracic X-Ray | EKG: SB <50bpm, pacemaker, RBBB, LAHB + RBBB or nonspecific intraventricular delay)  EchoC: left ventricular diastolic diameter >56 mm and/or ejection fraction <50%). | No classified | RBBB or RBBB+LAHB: 21 Pacemaker: 6 Other: 14 |
| **Carrasco M**. Et al 2003 Chile | EKG + EchoC | EKG: conductive alterations, arrythmias or pacemaker  Clinical: heart failure | No classified | Conductive alterations: 32  Cardiomyopathy: 19  Pacemakers: 9 |
| **Zulantay I.** Et al 2005 Chile | EKG | Not specified | No classified | LAHB: 4  Extended QT: 1  Incomplete RBBB: 1  Nodal rhythm: 1  Incomplete RBBB + LAHB: 1  RBBB + LAHB: 1  Atrio-ventricular block + LAHB: 1 |
| **Borges-Pereira J.** Et al 2008 Brazil | EKG | Minessota code classification system*^1^ | No classified | Frequent ventricular extrasystole: 3  RBBB + LAHB: 1  Complete atrio-ventricular block: 1  Altered repolarization: 1 |
| **Murcia L.** Et al 2010 Spain | EKG + EchoC + Thoracic X-Ray | Kuschnir criteria*^2^ | No classified | No specified |
| **Sabino EC.** Et al 2015 Brazil | EKG + EchoC | Minessota code classification system*^1^ | No classified | RBBB: 44  Pathological Q waves: 21  Isolated ST-T abnormalities: 21  Frequent ventricular extrasystole: 13  Atrial fibrillation: 3  Pacemaker: 21  LAHB: 41  Abnormal wall motion: 86  Reduced LVEF <50%: 85 |
| **Kaplinsky M**. Et al 2015 Bolivia | EKG | EKG: RBBB, LAHB, LPHB, ventricular extrasystoles, atrio-ventricular blocks, SB <45bpm, atrial fibrillation, junctional rhythm.  Incomplete RBBB was excluded | No classified | RBBB: 12  LAHB: 6  LPHB: 2  Ventricular extrasystoles: 1  SB: 5  Atrio-ventricular block: 3  Atrial ectopic rhythm: 4  Junctional scape rhythm: 2  More than 1 abnormalities: 28 |
| **Apt W.** Et al 2016 Chile | EKG + EchoC | NYHA criteria*^3^ | NYHA 1: 44 NYHA 2: 66 | Auricular arrythmia: 51  Ventricular arrythmia: 3  Atrio-ventricular block: 9  Intraventricular blocks: 35  Ischemia image: 5  Repolarization alteration: 5  Hypertrophic pattern 6  Prolonged QTc interval 19 |
| **Sánchez-Montalvá A.** Et al 2017 Spain | EKG + EchoC + Thoracic X-Ray | EKG: RBBB, LAHB, LPHB, LBBB, ventricular extrasystole, Q waves, ST-T changes, atrio-ventricular blocks, SB <50 bpm, atrial fibrillation or flutter, and pacemaker rhythm.  Kuschnir criteria*^2^ and Minessota code classification system*^1^ | Kuschnir 1: 98 Kuschnir 2: 8 Kuschnir 3: 9 Isolated EchoC alteration: 27 Cardiomegaly: 10 | SB: 23  Atrial fibrillation: 3  Atrial extrasystole: 5  Atrio-ventricular block: 17  QTc impairment: 15  RBBB 27  LAHB: 80  LPHB: 3  LBBB: 1  Ventricular extrasystole: 2  Left ventricular hypertrophy:3  Low voltage: 9  T-ST alterations: 9  Q wave: 2 |
| **D'Ávila D.** Et al 2018 Brazil | EKG + EchoC + Thoracic X-Ray | Rocha criteria*^4^ | CCC1: 3 CCC2: 4 CCC3: 16 CCC4: 7  CCC5: 38 | No specified |
| **Salvador F.** Et al 2020 Spain | EKG + Thoracic X-Ray | Kuschnir criteria*^2^ | Kuschnir 1: 8 Kuschnir 2: 1 Kuschnir 3: 2 | Only 7 patients specified:  RBBB: 4  LAHB: 2  LPHB: 1 |
| **Imai K.** Et al 2019 Japan | EKG + EchoC | CCC: 4 | No specified | Left heart failure: 3  Arrythmia: 1 |

Abbreviations: bpm: beat per minute; CCC: chronic chagasic cardiomyopathy; EchoC: echocardiography; EKG: electrocardiogram; LAHB: left anterior hemiblock; LPHB: left posterior hemiblock; LVEF: left ventricle ejection fraction; PCR: polymerase chain reaction; RBBB: right bundle branch block; SB: sinus bradycardia.

*^1^: Minnesota code classification is a method used for EKG interpretation unspecific for CD. *Prineas, RJ.Crow, RS., Blackburn, H., editors. The Minnesota Code Manual of electrocardiographic Findings. Littleton, MA: John Wright-PSG; 1982.*

*^2^: Group 0: normal EKG and chest X-Ray; Group 1: abnormal EKG without cardiac enlargement; Group 2: abnormal EKG and cardiac enlargement without clinical signs of heart failure; Group 3: heart failure.

*^3^: New York Heart Association classification for heart failure: NYHA I: structural myocardial changes, NYHA II: small decrease in exercise tolerance; NYHA III: significant decrease in exercise tolerance; NYHA IV: symptoms of heart failure in rest or during small exercise.

*^4^: CCC1: minimal alteration on EchoC or Holter; CCC2: NYHA1 and minor EKG findings: : low voltage, LHAB, minor ST changes…; CCC3: NYHA 2 and EKG findings: RBBB or ventricular extrasystole; CCC4: significant EKG alterations: RBBB + LHAB, Q waves, ST alterations, LBBB, atrio-ventricular block; CCC5: cardiac enlargement

**Figure Appendix 5: QUIPS summary**

**Appendix 6: Detailed QUIPS assessment by study**

| **Reference** | **Comments** |
| --- | --- |
| **Salomone** 2000 | Study participation: consecutive patients. Correct diagnosis method. However, no inclusion period specification.  Study attrition: no missing participants.  Prognostic factor measurement: sample collection and characteristics are well defined. PCR method is described. No cut-off values specified.  Outcome measurement: CCC criteria well described but they don’t use a standardized one. No individual characteristics are collected.  Study confounding: there is no description or exclusion of possible cardiovascular risk confounders.  Statistical analysis and reporting: data is well presented, and statistical analysis is appropriate. |
| **Carrasco M**. 2003 | Study participation: consecutive patients. Correct diagnosis method, recruitment period and site are well specified.  Study attrition: several patients are lost before PCR determination. No information on their characteristics or reason for their loss of follow-up.  Prognostic factor measurement: Sample collection and PCR method is well described. No cut-off values or internal/external controls are described.  Outcome measurement: Although CCC assessment is well described; there is neither specific classification nor individual outcomes.  Study confounding: there is no description or exclusion of possible cardiovascular risk confounders.  Statistical analysis and reporting: data and statistical analysis is well presented. However, both cardiac and digestive involvements are analyzed together. |
| **Zulantay I.** 2005 | Study participation: there is no description about recruitment process or period.  Study attrition: no specification about loss to follow-up or patient characteristics.  Prognostic factor measurement: accurate methodology description. Internal and external controls with 100% correlation.  Outcome measurement: CCC description and classification well described. Blind EKG reading by two investigators.  Study confounding: there is no description or exclusion of possible cardiovascular risk confounders.  Statistical analysis and reporting: No clear reporting. Indirect data is used. Statistical analysis is not designed for the purpose of this review. |
| **Borges-Pereira J.** 2008 | Study participation: seroprevalence study. Recruitment methodology and period are well defined.  Study attrition: there are few patients lose and they are well described with no external validation compromise.  Prognostic factor measurement: PCR measurement is well described. Sample collection and conservation is explained. No external controls for methodology validation.  Outcome measurement: CCC description and classification well described. Blind EKG reading by two investigators.  Study confounding: there is no description or exclusion of possible cardiovascular risk confounders  Statistical analysis and reporting: no clear reporting. Indirect data used. Statistical analysis is not designed for the purpose of this review. |
| **Murcia L.** 2010 | Study participation: cohort patients for a specific Chagas unit. Recruitment methodology and period are well defined.  Study attrition: no missing participants.  Prognostic factor measurement: Sample collection and PCR method is well described. Internal controls are described.  Outcome measurement: CCC assessment and classification is well described. No reporting of individual results. No double EKG evaluation.  Study confounding: there is no description or exclusion of possible cardiovascular risk confounders.  Statistical analysis and reporting: data is well presented, and statistical analysis is appropriate. |
| **Sabino EC.** 2015 | Study participation: retrospective analysis of recruited patients from blood bank and CCC clinic. Period is defined. No information about CCC patient recruitment.  Study attrition: there are few patients loses and they are well described with no external validation compromise  Prognostic factor measurement: accurate methodology description. Internal and external controls are used.  Outcome measurement: CCC description and classification well described. Blind EKG reading by three investigators. Individual data is presented.  Study confounding: Although there is no stratified analysis, main possible cardiovascular confounders are excluded.  Statistical analysis and reporting: data is well presented, and statistical analysis is appropriate. |
| **Kaplinsky M**. 2015 | Study participation: only child-bearing women included. Recruited process is well described and period is specified.  Study attrition: PCR is only available for a small group of participants.  Prognostic factor measurement: well defined methodology but PCR method changed ad mid-term of the study.  Outcome measurement: CCC description and classification well described. Blind EKG reading by two investigators. Individual data is presented.  Study confounding: there is no description or exclusion of possible cardiovascular risk confounders  Statistical analysis and reporting: indirect data used. Statistical analysis is not designed for the purpose of this review. |
| **Apt W.** 2016 | Study participation: randomly selected patients from a representative population. Well described process and period.  Study attrition: no missing participants.  Prognostic factor measurement: Sample collection and PCR method is well described. Standardized method is used. Internal and external controls are described.  Outcome measurement: CCC description and classification well described. Blind EKG reading by two investigators. Individual data is presented.  Study confounding: Although there is no stratified analysis, main possible cardiovascular confounders are excluded.  Statistical analysis and reporting: indirect data used. Statistical analysis is not designed for the purpose of this review. |
| **Sánchez-Montalvá A.** 2017 | Study participation: consecutive patients from a representative population. Period and recruitment site is well defined.  Study attrition: no missing participants.  Prognostic factor measurement: Sample collection and PCR method is well described. Standardized method is used. Internal controls are described.  Outcome measurement: CCC description and classification well described. Blind EKG reading by two investigators. Individual data is presented  Study confounding: A detailed list of possible cardiovascular confounders is excluded.  Statistical analysis and reporting: indirect data used. Statistical analysis is not designed for the purpose of this review |
| **D'Ávila D** 2018 | Study participation: patient recruitment from a Chagas disease clinic. No period or methodology specified.  Study attrition: no missing participants.  Prognostic factor measurement: Sample collection and PCR method is well described. Standardized method is used. Internal controls are described.  Outcome measurement: CCC assessment and classification is well described. No reporting of individual results.  Study confounding: there is no description or exclusion of possible cardiovascular risk confounders.  Statistical analysis and reporting: indirect data used. Statistical analysis is not designed for the purpose of this review |
| **Salvador F.** 2020 | Study participation: consecutive patients from a representative population. Period and recruitment site is well defined.  Study attrition: no missing participants.  Prognostic factor measurement: Sample collection and PCR method is well described. Standardized method is used. Internal and external controls are described  Outcome measurement: CCC assessment and classification is well described. No reporting of individual results.  Study confounding: there is no description or exclusion of possible cardiovascular risk confounders  Statistical analysis and reporting: indirect data used. Statistical analysis is not designed for the purpose of this review |
| **Imai K.** 2019 | Study participation: patients directly selected after suspicion of organ involvement.  Study attrition: No missing participants.  Prognostic factor measurement: There is scarce information on PCR method or using of internal control.  Outcome measurement: CCC classification is not well described. No standardized classification used. No individual data.  Study confounding: there is no description or exclusion of possible cardiovascular risk confounders  Statistical analysis and reporting: Very small sample. No clear data. Statistical analysis is not designed for the purpose of the review. |
