# Supplementary figures and images for: Association Between Trypanosoma cruzi DNA in Peripheral Blood and Chronic Chagasic Cardiomyopathy: A Systematic Review
Source: Front Cardiovasc Med. 2022 Jan 31;8:787214. doi: 10.3389/fcvm.2021.787214 (PMC8841718; doi:10.3389/fcvm.2021.787214)

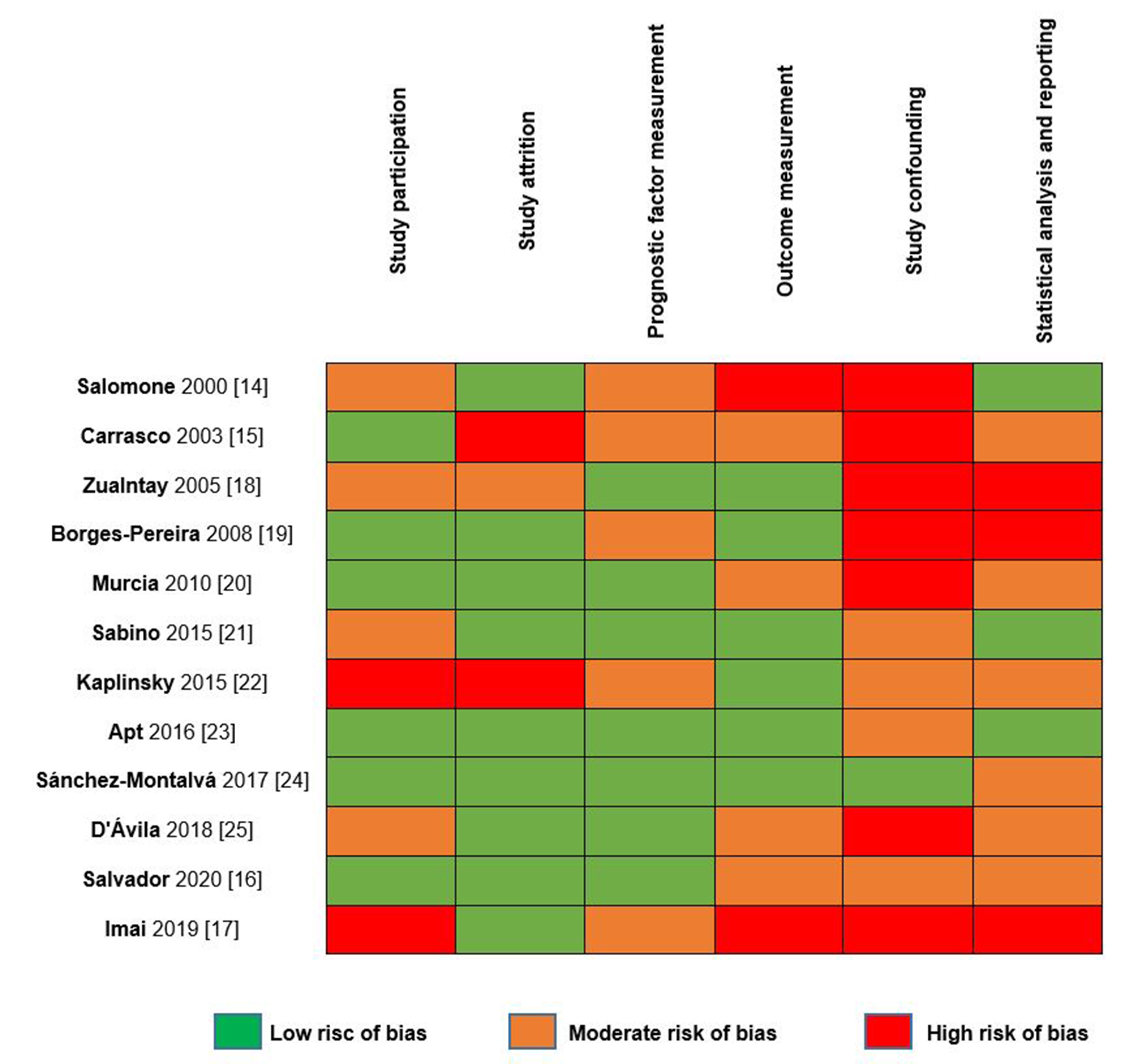

Supplement: Supplementary file 2 [file Image_1.TIF]
